# Neurophysiological Aspects of REM Sleep Behavior Disorder (RBD): A Narrative Review

**DOI:** 10.3390/brainsci11121588

**Published:** 2021-11-30

**Authors:** Michela Figorilli, Giuseppe Lanza, Patrizia Congiu, Rosamaria Lecca, Elisa Casaglia, Maria P. Mogavero, Monica Puligheddu, Raffaele Ferri

**Affiliations:** 1 Neurology Unit, Department of Medical Sciences and Public Health, University of Cagliari and AOU Cagliari, Monserrato, 09042 Cagliari, Italy; michelafigorilli@gmail.com (M.F.); patcongiu@gmail.com (P.C.); rosamaria.lecca87@gmail.com (R.L.); elisa.casaglia@outlook.com (E.C.); puligheddu@unica.it (M.P.); 2 Sleep Disorders Center, Department of Medical Sciences and Public Health, University of Cagliari, Asse Didattico E., SS 554 Bivio Sestu, Monserrato, 09042 Cagliari, Italy; 3 Clinical Neurophysiology Research Unit, Oasi Research Institute-IRCCS, Via Conte Ruggero 73, 94018 Troina, Italy; glanza@oasi.en.it; 4 Department of Surgery and Medical-Surgical Specialties, University of Catania, Via Santa Sofia 78, 95123 Catania, Italy; 5 Istituti Clinici Scientifici Maugeri, IRCCS, Scientific Institute of Pavia, 27100 Pavia, Italy; paola_mogavero@libero.it

**Keywords:** REM sleep behavior disorder, REM sleep without atonia, polysomnography, neurophysiology, electroencephalography, transcranial magnetic stimulation, vestibular evoked myogenic potentials

## Abstract

REM sleep without atonia (RSWA) is the polysomnographic (PSG) hallmark of rapid eye movement (REM) sleep behavior disorder (RBD), a feature essential for the diagnosis of this condition. Several additional neurophysiological aspects of this complex disorder have also recently been investigated in depth, which constitute the focus of this narrative review, together with RSWA. First, we describe the complex neural network underlying REM sleep and its muscle atonia, focusing on the disordered mechanisms leading to RSWA. RSWA is then described in terms of its polysomnographic features, and the methods (visual and automatic) currently available for its scoring and quantification are exposed and discussed. Subsequently, more recent and advanced neurophysiological features of RBD are described, such as electroencephalography during wakefulness and sleep, transcranial magnetic stimulation, and vestibular evoked myogenic potentials. The role of the assessment of neurophysiological features in the study of RBD is then carefully discussed, highlighting their usefulness and sensitivity in detecting neurodegeneration in the early or prodromal stages of RBD, as well as their relationship with other proposed biomarkers for the diagnosis, prognosis, and monitoring of this condition. Finally, a future research agenda is proposed to help clarify the many still unclear aspects of RBD.

## 1. Introduction

For the *International Classification of Sleep Disorders 3rd Edition* (ICSD-3) [1], rapid eye movement (REM) sleep behavior disorder (RBD) is a parasomnia manifested by vivid, often frightening dreams accompanied by simple or complex motor behaviors during REM sleep. Patients seem to “enact their dreams” with their behaviors, probably mirroring the dream content [2]. Idiopathic (or isolated) RBD (iRBD) is an established early manifestation of a neurodegenerative disease, especially synucleinopathy [3]. RBD can be found in a large proportion of both children and adults with narcolepsy, representing a form of REM sleep motor behavior dyscontrol [4,5], and it seems to be a phenotype distinct from iRBD, with less marked sex predominance, more elementary and less complex movements and less violent behavior in REM sleep, younger age of onset, and orexin deficiency (a feature of narcolepsy type 1) [6,7].

The polysomnographic (PSG) hallmark of RBD is decreased muscle atonia during REM sleep, also called REM sleep without atonia (RSWA), because of increased electromyographic (EMG) activity during this stage, and this is a feature essential for the diagnosis of this condition, which is thus based on both clinical and laboratory findings [1]. The search for reliable biomarkers of RBD and its evolution into an overt synucleinopathy is a very lively field of interest, with many studies being published [8]. However, RSWA remains the most important neurophysiological aspect, being necessary for the diagnosis of RBD [9]. In addition, several additional neurophysiological aspects have recently been investigated in depth, which will constitute the focus of this narrative review together with RSWA.

## 2. REM Sleep Network and REM Atonia Neurophysiopathology

Knowledge about the neural circuitry underling REM sleep physiology and mechanisms causing RSWA mostly arises from animal, post-mortem, and radiological studies. Brainstem lesions due to neurodegeneration, demyelination, tumors, or ischemic injury may cause RSWA and lead to secondary RBD [10,11,12,13].

Early animal studies showed that a state characterized by muscle atonia and rapid eye movement persists following decortication, or brainstem transections rostral to the pons in Jouvet’s “pontine cat” [14]. In addition, the sublaterodorsal tegmental nucleus (SLD), also called the subcoeruleus nucleus (SubC), contains many neurons that show tonic firing selective to the paradoxical (or REM) sleep (PS) state, thus called “PS-on” neurons [15]. Although initial studies indicated that the nature of SLD or SubC PS-on neurons was cholinergic [16], further research demonstrated that vesicular glutamate transporter 2 (vGlut2), a specific marker of glutamatergic neurons, was expressed in c-Fos-positive neurons localized in the SLD or SubC after PS hypersomnia [17]. Moreover, genetic inactivation of glutamatergic transmission in SLD or SubC neurons induces a 30% decrease in PS quantities and the occurrence of RBD, confirming the glutamatergic nature of SLD or SubC neurons generating PS [18]. Tract-tracing data also revealed that SLD or SubC glutamatergic PS-on neurons send descending projections to the gamma-amyno-butyric acid (GABA) and glycinergic neurons located in the nucleus raphe magnus, as well as the ventral alpha gigantocellular and lateral paragigantocellular reticular nuclei, inducing muscle atonia, but not to the intralaminar thalamus neurons known to mediate the activation of the cortex during REM sleep [18]. Conversely, cholinergic and noncholinergic neurons, located in the SLD or SubC, pedunculopontine, and laterodorsal tegmental nuclei, and glutamatergic neurons in the reticular formation projecting to the thalamus and hypothalamus (that seem to be firing both during waking and PS) are believed to play a role in cortical activation during REM sleep. Concerning the limbic cortical structures, only the dentate gyrus, anterior cingulate, and retrosplenial and medial entorhinal cortices seem to be activated during PS [19].

Recent findings suggest that the onset of a REM sleep episode might be due to a complex and still largely unknown mechanism implicating the activation of PS-on melanin-concentrating hormone and GABAergic neurons in the lateral hypothalamus [20]. This would remove the GABAergic tone of PS-off neurons located in the ventrolateral part of the periaqueductal gray (vlPAG) and the adjacent dorsal part of the deep mesencephalic nucleus (dDPMe), which gate PS by tonically inhibiting the PS-on neurons of the SLD or SubC during wakefulness and slow-wave sleep [21,22], combined with the continuous presence of a glutamatergic input. PS-on GABAergic neurons localized in the lateral hypothalamus, dorsal paragigantocellular nuclei, and vlPAG also seem to inactivate the PS-off orexin and aminergic neurons during REM sleep [23]. Conversely, the activation of arousal systems, reciprocally inhibiting the GABAergic PS-on neurons, might determine the exit from a REM sleep state [19], allegedly forming a “flip-flop” switch model for REM sleep.

**Figure 1:**
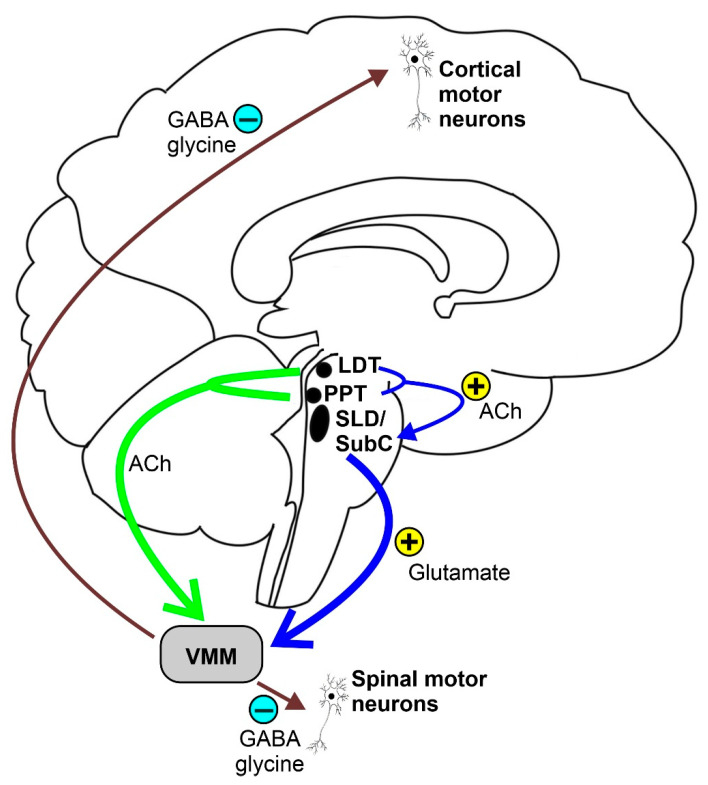
Schematic representation of the REM sleep atonia circuitry. Ach = acetylcholine, LDT = laterodorsal tegmental nucleus, PPT = pedunculopontine nucleus, SLD = sublaterodorsal tegmental nucleus, SubC = subcoeruleus nucleus, and VMM = ventromedial medulla.

REM sleep atonia (see Figure 1) is basically due to both the inhibition and decreased activation [24] of cranial and spinal skeletal motor neurons, which exhibit a tonic hyperpolarization during physiological REM sleep [25]. In fact, blockage of both the GABA and glycine motor neuron receptors prevents muscle paralysis during REM sleep [26]. During REM sleep, cranial and spinal motor neurons are very likely to be inhibited by the GABA and glycinergic neurons located in the ventromedial medulla (VMM). Additionally, the spinal motor neurons seem to also be inhibited by the spinal interneurons [26,27]. Moreover, decreased or lost glutamatergic, noradrenergic, dopaminergic, and hypocretinergic activity may contribute to reduce motor neuron excitability, thus strengthening muscle atonia during REM sleep [28,29,30]. The VMM and spinal interneurons are under the direct control of a group of glutamatergic neurons located within a small reticular region of the pontine tegmentum, namely the SLD or SubC, which act as an REM sleep generator and controller (REM-on cells), which is thought to be necessary and sufficient for generating REM sleep atonia [13,31,32,33]. SLD or SubC glutamatergic REM-on cells and VMM GABA and glycinergic neurons constitute the main REM atonia circuitry [34].

Some studies suggest a cholinergic modulation of REM paralysis circuitry [35]. Weng et al. [36] showed that SLD neurons are influenced by acetylcholine, which was found to exert both pre-and post-synaptic excitatory effects on glutamatergic spinally projecting SLD neurons. These cells should promote REM atonia, suggesting a cholinergic mechanism of regulation of REM sleep atonia. SLD neurons are likely to be activated by cholinergic neurons of the pedunculopontine and laterodorsal tegmental nuclei during REM sleep.

The first animal model of RBD was described by Michael Jouvet [14], who found that physical lesions of the dorsal pontine tegmentum interneurons caused abnormal motor behavior during REM sleep in cats, mimicking those observed in human RBD. Subsequent animal models showed that the generator of atonia was located in the SLD and VMM nuclei, which act as an integral brainstem circuit, playing a crucial role in triggering physiological REM sleep atonia. They also showed that the glutamatergic neurons within the SLD are necessary and sufficient for generating REM atonia. Accordingly, selective damage to glutamatergic SLD projections causes RSWA [13,34,37]. Conversely, stimulation of these regions during wakefulness produced muscle paralysis [33]. Postmortem analysis of brains from patients with iRBD showed neural loss, gliosis, Lewy bodies, and neurite deposition in the brain areas involved in controlling REM sleep, such as SLD and VMM [13,38]. Moreover, signs of damage in the dorsal pons and the subcoeruleus region in patients with RBD were also shown by neuroimaging studies [39,40].

In conclusion, the SLD nucleus, and namely its glutamatergic REM-on neurons, is the brain region that triggers REM sleep atonia by stimulating the VMM and the spinal interneurons that directly inhibit motor neurons by GABA-and glycinergic projections. SLD and VMM constitute the so-called “REM sleep atonia circuit”, and lesions in this area prevent REM sleep paralysis.

## 3. REM Sleep without Atonia (RSWA)

### 3.1. Polysomnographic Features

Physiological REM sleep is characterized by complete muscle atonia with a markedly decreased amplitude for the EMG signal. As said above, RSWA is the neurophysiological and polysomnographic hallmark of RBD, and it is represented by a persistent muscle tone during REM sleep, resulting in either sustained (tonic) excessive activity during REM sleep in the chin EMG, intermittent (phasic) excessive activity during REM sleep in the chin or limb EMG, or both. The tonic and phasic components of RSWA can be present and isolated from each other or in combination, such as bursts of phasic EMG activity superimposed on a tonically increased muscle tone. According to the latest American Academy of Sleep Medicine (AASM) scoring manual [41], a 30-s epoch of REM sleep is defined as tonic when at least 50% of its duration contains a chin EMG with an amplitude greater than the minimum amplitude observed in non-REM sleep. On the other hand, the same scoring manual defines the phasic components of RSWA as excessive transient muscle activity bursts on the chin and limb EMG channels lasting 0.1–5.0 s with an amplitude at least 4 times as high as the background EMG activity. Moreover, a 30-s REM sleep epoch, subdivided into 3-s mini-epochs, is considered to have excessive phasic EMG activity when at least 50% of the 3-s mini-epochs contain bursts of transient EMG activity [41].

RSWA must be differentiated from increased muscle tone related to other different activities (artifacts), such as respiratory arousals, snoring, limb movements, and body movements. Furthermore, RSWA can be triggered or exacerbated by different drugs, such as tricyclic antidepressants, selective serotonin reuptake inhibitors, monoamine oxidase inhibitors, mirtazapine, venlafaxine, and betablockers. However, further studies are needed to assess cause–effect relationships [42,43,44,45,46,47]. Although the quantification of RSWA is mandatory for the diagnosis of RBD, both the AASM scoring manual and the ICSD-3 do not provide established cut-off values for RSWA, nor do they say how many tonic phasic epochs are needed to define RSWA [1,41].

### 3.2. Visual Scoring

Different visual scoring methods to detect and quantify RSWA have been developed and validated. However, there is no consensus on which method is more efficient or which muscle group is better suited to identify or quantify RSWA and distinguish RBD from healthy subjects. It is important to note that in this paper, we refer to phasic or tonic EMG activations during REM sleep that should not be confused with the phasic or tonic subtypes of REM sleep, usually identified on the basis of the presence or absence of eye movement activity during this sleep stage.

The first and the most widely used visual method for scoring RSWA was developed in 1992 by Lapierre and Montplaisir [48], here referred to as the “Montreal” method. According to this method, a 20-s REM sleep epoch is defined as tonic when sustained chin EMG lasts more than 50% of the epoch duration, with an amplitude at least twice the background EMG activity or greater than 10 µV. The percentage of tonic REM sleep epochs defines the tonic EMG density. Phasic activity is defined as bursts of EMG activity with an amplitude exceeding 4 times the background and lasting 0.1–10 s. Phasic EMG activity can occur in either atonic or tonic REM sleep epochs and is scored by dividing a 20-s [48] or a 30-s [49] REM sleep epoch into 2-s mini-epochs. The percentage of 2-s mini-epochs containing phasic chin EMG activity defines the phasic chin EMG density. The authors validated this method in a population of 80 subjects with clinical diagnoses of RBD, identifying RSWA if there was a tonic EMG density ≥30% or phasic chin EMG density ≥15% [49,50].

The Barcelona Innsbruck group (SINBAR) validated their visual scoring methods in 30 RBD patients (15 iRBD and 15 associated with Parkinson’s Disease (PD)) and 30 control subjects, analyzing REM sleep EMG activity in 11 different muscles, including the mentalis muscle and the flexor digitorum superficialis muscle (FDS), bilaterally in the upper limbs [51]. Tonic chin EMG activity was scored in 30-s epochs only in the mentalis muscle according to the Montreal method [50]. Phasic EMG events were scored separately in each EMG channel in 3-s mini-epochs as bursts of EMG activity lasting 0.1–5 s with an amplitude at least twice the background. Each 3-s mini-epoch was scored as having or not having phasic EMG activity. When phasic EMG activity bursts emerged in 3-s mini-epochs with tonic EMG activity, the amplitude of the phasic activity had to be at least twice the tonic background EMG activity. Moreover, each 3-s mini-epoch was scored as having or not having any EMG activity, containing irrespectively tonic or phasic activity or a combination of them [51]. The SINBAR group suggested the following cut-off values with the best specificity and sensitivity to identify RBD: >16.3% of the 3-s mini-epochs containing phasic chin EMG activity, >18% of the 3-s mini-epochs having any chin EMG activity, >32% of the 3-s mini-epochs having any chin EMG activity combined with bilateral FDS phasic EMG activity, and >27% of the 30-s epochs having any chin EMG activity combined with bilateral FDS phasic EMG activity [51]. The latter cut-off value was suggested as the most reliable evidence-based cut-off to distinguish RBD patients from their controls in the ICSD-3 [1].

Another group developed a visual scoring method for short-duration phasic EMG activity during REM sleep, known as the phasic EMG metric (PEM), in the submentalis, tibialis anterior, and brachioradialis muscles for the identification of RBD [52]. The PEM was defined as the percentage of 2.5-s intervals containing any discrete bursts of EMG activity lasting ≥100 ms with an amplitude at least 4 times higher than the pre-sleep background activity. The RBD patients showed higher levels of PEM activity in the mentalis and brachioradialis muscles than their controls [52].

Another visual scoring method was developed by McCarter et al. [53] by combining the tonic, phasic, and any EMG activity in the submentalis and tibialis anterior muscles, which was validated in a cohort of 20 RBD patients with PD, 20 patients with obstructive sleep apnea, and 20 patients with snoring. The tonic EMG activity was scored in each 30-s REM sleep epoch according to the Montreal method [50]. Phasic and any EMG activity were scored in 3-s mini-epochs and identified as any activity lasting 0.1–14.9 s with an amplitude 4 times greater than the background [53]. The authors provided the following cut-off values for the definition of RSWA using 3-s mini-epochs: >15.5% phasic, 21.6% any, and 1.2% tonic submentalis EMG activity; 30.2% for phasic and any tibialis anterior EMG activity; and 37.9% phasic and 43.4% any EMG activity in the combined submentalis and tibialis anterior muscles [53]. However, the same authors further validated their method in a population of 45 consecutive patients, including 15 with iRBD, finding very similar RSWA diagnostic cut-off values [54].

Recently, a comparative study has shown a very high degree of agreement between the two visual scoring methods (i.e., the Montreal and SINBAR methods) when considering tonic or any EMG activity in a large cohort of PD patients with and without RBD [55].

Further larger studies are needed to assess a reliable and usable visual scoring method for quantifying RSWA in RBD or both iRBD and association with alpha-synucleinopathies and to assess which muscle groups or combinations of them are most reliable to identify RSWA.

Table 1 reports a short list of studies in which data are reported on the accuracy of some different methods for the quantification of RSWA being used for the diagnosis of RBD. Even if a relatively wide range of accuracy values have been reported with all visual methods, there seems to be a good agreement that all of them can perform reliably and with comparable degrees of accuracy.

### 3.3. Automatic Scoring

Visual scoring of RSWA is a time-consuming process and may be challenging even for expert scorers. Therefore, multiple efforts have been made to develop computerized methods for the quantification and detection of RSWA [65,66].

The method that, to date, counts the highest number of published articles is the so-called REM Atonia Index (RAI), proposed by Ferri et al. in 2008 [60,61,67]. It is based on the analysis of the amplitude of the rectified submentalis EMG signal in 1-s mini-epochs. Theoretically, the RAI varies from 0 (total loss of atonia) to 1 (complete atonia), and the threshold for definite RSWA is below 0.8 [61]. This method has shown a relatively low night-to-night variability [59], good sensitivity and specificity, and good correlation to the visual methods [49]. The RAI was validated for patients with iRBD [61] and PD-RBD [55,68], and it is also reliable in patients with RBD and comorbid OSA [54] and for the detection of RSWA in narcolepsy [69,70], including children [4].

In 2007, Burns et al. [71] developed an algorithm comparing the variance of the chin EMG during 3-s REM sleep mini-epochs to a threshold based on the variance during non-REM sleep, defining the supra-threshold REM EMG activity metric (STREAM) as the percentage of REM sleep mini-epochs above that threshold.

Mayer et al. [72] proposed a method for the quantification of motor activity over a baseline of clearly absent EMG chin activity during REM: each activation above twice the baseline is classified into short (sMI) and long movements (lMI), and an hourly index is calculated. This method demonstrated a sensitivity of 72.5% and a specificity of 86.7% for the detection of RBD. Kempfner et al. [73] used a support vector machine that, through a complex combination of subject-specific features and the detection of outliers, analyzed the chin and tibialis EMG and electrooculography signal and correctly separated patients with RBD, periodic leg movements, and healthy controls. The method proposed by Frandsen et al. [74] quantifies muscle activation. After an amplitude curve is generated, the median amplitude is calculated for each 30-s epoch. Muscle activity is identified when the curve is larger than four times a defined baseline.

Three algorithms published in the literature are based on the transcription of the visual criteria described in the previous paragraph: the SINBAR criteria [51] were applied in integrated software [63], the McCarter et al. criteria [53] were used in another study in 2018 [75], and Bliwise et al.’s PEM [52] was computed with the use of wavelet analysis [76]. Another semi-automatic algorithm was described in a small study, consisting of a complex processing analysis of the submentalis and tibialis EMG and electrocardiographic (ECG) channels [77].

Cooray et al. [78] correctly identified patients with RBD using a trained random forest classifier that combined the RAI [60,61], STREAM [71], and the method by Frandsen et al. [74] with the analysis of features of sleep architecture and the EMG fractal exponent. This method was reported to be feasible even with a minimum set of sensors (EMG and ECG) [79]. Recently, Cesari et al. [80] developed a data-driven method for the identification of movements in chin and tibialis anterior EMG using a probabilistic model of atonia in healthy controls and EMG areas that were not likely atonic, showing an accuracy of 70.8% for the identification of iRBD. In 2021, a similar data-driven system was implemented for the identification of PD-RBD [81].

All automatic methods described here have been reported to have acceptable sensitivity and specificity in their studies of presentation. However, their comparison showed variable performance, making it impossible to assess the optimal method [82], although the RAI seemed to be the most reliable in the identification of RBD. Further comparative studies on larger numbers of recordings are required to identify the most reliable algorithm in order to introduce it in the scoring guidelines for everyday clinical practice. However, as reported in Table 1, automatic methods seem to perform with an accuracy comparable to that of visual methods.

## 4. Other Neurophysiological Features

### 4.1. Electroencephalography during Wakefulness and Sleep

Patients with iRBD show some electroencephalographic (EEG) features that have also been reported in neurodegenerative disorders, such as PD and dementia with Lewy bodies (DLB) [83]. These changes typically include diffuse EEG slowing during wakefulness, although this is more evident over the occipital scalp areas [84]. Notably, this cortical slowdown has been associated with cognitive decline, thus suggesting a parallel between neuropsychological and electrophysiological impairment. It should be remarked that higher absolute delta and theta power in all cortical regions were able to identify iRBD patients with a higher risk of short-term progression into alpha-synucleinopathy [84].

EEG-derived sleep macro- and microstructure changes have also been proposed as neurophysiological markers. However, despite some intriguing findings, no longitudinal studies have been carried out. A previous study only reported that RSWA quantification, particularly high tonic chin EMG activity at the baseline, could predict a higher risk of conversion to PD [84]. A recent high-density EEG study revealed abnormal sleep homeostasis in iRBD patients [85]. Namely, the healthy controls (but not the RBD patients) exhibited a decrease in beta power during phasic compared with tonic REM sleep. Moreover, the RBD patients displayed a reduced decline in SWA from early to late non-REM sleep, as well as reduced overnight changes in the distribution of the slow-wave amplitude. Therefore, without suppression of the beta rhythms during phasic REM sleep, RBD patients showed increased cortical arousal, possibly favoring the occurrence of behavioral episodes. The authors concluded that a blunted difference between the REM sleep substages may represent a sensitive biomarker for RBD and that a reduced overnight decline in SWA would suggest a reduced synaptic plasticity in RBD [85]. Eventually, this would favor progression toward a neurodegenerative disorder.

Another study indicated the presence of increased EEG microstructure instability during REM sleep in drug-naïve iRBD patients [86], with power frequencies of the EEG spectrum <15 Hz showing a smaller REM-related decrease than the controls and an increase in the beta band (possibly correlated with the persistence of muscle activity). Treatment with clonazepam induced a partial return of the power of frequencies <15 Hz to normal values. Instability of the normalized EEG power was also increased and was significantly decreased by clonazepam.

Studies on the cyclic alternating pattern (CAP) [87] assessing non-REM sleep instability have also been carried out with few consistent results. One report found increased slow EEG transients (called A1 CAP subtypes) and decreased fast EEG events (A2 and A3 CAP subtypes) in untreated iRBD patients [88]. In the same study, long-term treatment with clonazepam was found to be accompanied by a decrease in non-REM sleep instability and the duration of EEG transients. Another study reported different findings, with a global increase in the CAP due in particular to the A2 and A3 subtypes. On the contrary, the A1 CAP subtypes were found to be decreased in RBD patients [89]. Finally, a third study found decreased global CAP measures in iRBD patients, especially because of a decrease in A1 CAP subtypes [90]. Thus, the value of the study of non-REM sleep instability in RBD still remains to be clarified.

Additionally, the sleep spindle density has been reported to be abnormal in patients with RBD, with a decrease in fast (but an increase in slow) spindles, which was interpreted as a potentially predictive biomarker of future neurodegeneration in these patients [91]. Another study also reported a global decrease in the spindle density in iRBD and PD patients with RBD and suggested that this decrease might be considered a supportive diagnostic tool in these conditions [92].

It is also worth mentioning a recent study based on advanced EEG analysis methods reporting that theta-band bursts and a decreased alpha-band burst in EEG, recorded during wakefulness with eyes closed, might predict future conversion to PD or DLB [93]. Finally, artificial intelligence and machine learning, combining information on muscle atonia and sleep architecture, promise to be able to perform an accurate identification of RBD patients in the near future [78].

Finally, recent studies have suggested that the use of information derived from the electrooculogram, a necessary signal for sleep staging, can be useful for supportting the diagnosis of RBD, especially when associated with other parameters derived from EMG [79] or EEG [81] analysis.

### 4.2. Transcranial Magnetic Stimulation

Non-invasive brain stimulation techniques, such as transcranial magnetic stimulation (TMS) and transcranial direct current stimulation, are widely used to functionally investigate the neural pathways and brain network in vivo, also providing prognostic measures and neuromodulatory activity [94,95,96,97,98,99,100]. Accordingly, the data from EEG and functional neuroimaging, during both wakefulness and sleep, have suggested the involvement of different transmission systems which seem to be impaired in synucleinopathy, including PD, multiple system atrophy, and DLB [101,102].

Coherently, a number of studies have been carried out to evaluate the electrophysiological pattern of cortical excitability, neural plasticity, and functional connectivity to TMS in different sleep disorders [103,104,105,106]. In particular, most of the evidence converged on changes to both short-latency intracortical inhibition (SICI) and intracortical facilitation (ICF) in patients with PD, including those in the early stages, in terms of decreased SICI and reduced ICF. Translationally, this would indicate a disinhibition and a hypofacilitation of the motor cortex that are compatible with an impaired GABA and glutamate neurotransmission, respectively [107,108,109,110,111,112,113].

On the contrary, to date, only two TMS studies have been performed with iRBD patients. The first [114] reported an impairment of the short-latency afferent inhibition (SAI) that may support the hypothesis of cholinergic dysfunction in subjects developing cognitive impairment. This result was confirmed in a second study by the same research group on patients with RBD associated to PD, which can be viewed as the correlate of a cholinergic involvement at the basis of the cognitive decline observed in these subjects [115]. The authors concluded that cholinergic degeneration significantly contributes to non-motor Parkinsonian features, also raising the possibility that RBD increases the risk of cognitive impairment in PD [114]. Taken together, these findings may help to achieve an early recognition of the cognitive decline in PD and stimulate future “TMS-targeted” treatments [101]. The second very recent study [116] found that in patients still asymptomatic for a neurodegenerative disorder, changes in ICF and, to a lesser extent, SICI might precede the onset of future neurodegeneration. Furthermore, SICI correlated with the muscle tone alteration during REM sleep, possibly supporting the proposed RBD model of retrograde influence on the cortex from the brainstem [116].

Based on these considerations, it can be hypothesized that the activity of the brainstem centers may also be implicated in the physiological suppression of muscle activity during wakefulness [117]. Conversely, in RBD, an impaired control, arising from the brainstem and ascending to the supratentorial structures, might cause both the reduction of REM sleep atonia and an imbalance between SICI and ICF in favor of the former. A recent EEG study also seems to support this hypothesis. The REM sleep microstructure EEG changes indicate subtle but significant alterations in the cortical electrophysiology in isolated RBD patients, possibly representing the early stage of a future neurodegenerative process [86].

Overall, although still limited, TMS studies provide novel insights into the mechanisms underlying cortical dysfunction in PD and RBD and might open future therapeutic avenues. When integrated with clinical, neuroimaging, and sleep-related data, TMS findings are suggestive of an electrocortical imbalance with multiple neurotransmission pathways involved in RBD. Longitudinal studies are required to verify whether the abnormalities detected at this early stage do correlate with the clinical progression of RBD.

### 4.3. Vestibular Evoked Myogenicpotentials

The brainstem has been identified as a key player in the pathophysiological process leading to the development of iRBD. Vestibular evoked myogenic potentials (VEMPs) (cervical (cVEMP), masseter (mVEMP), and ocular (oVEMP) VEMPs) are a group of neurophysiological tools for the extensive and indirect assessment of the brainstem’s function along its whole length, thus also allowing the identification of subclinical brainstem abnormalities in clinically and radiologically silent regions [118].

De Natale et al. [119] were the first to report significantly altered VEMPs in iRBD patients (without any sign of motor impairment), with a primary and severe involvement of VEMPs assessing the rostral part of the brainstem (mVEMP and oVEMP), implying that cellular and connection derangement are more represented in this brainstem area, which is also in line with the current pathophysiological understanding of RBD in PD [120]. Furthermore, the correlation between RSWA and VEMP alteration might represent a prodromal aspect that anticipates the possible evolution from iRBD to neurodegeneration, while dopamine transporter scan alteration might represent a later step [121]. These findings enrich the knowledge on the degree of brainstem involvement in iRBD and constitute the basis for further research in this field, including follow-up studies to assess indirectly the evolution of VEMP abnormalities (as an example, to define a subpopulation of iRBD patients with or without VEMPs alterations who will or will not develop a synucleinopathy).

## 5. Role of Neurophysiological Assessment in the Study of RBD

### 5.1. Neurophysiology and Neurodegeneration

Overall, the electrophysiological evidence reviewed here converges on the hypothesis that RBD can modify the global neurochemical balance in areas of the central nervous system broader than the specific atonia-generating brainstem circuitry (i.e., the subcoeruleus nucleus and ventro-lateral medulla), also reaching the cortical level through some ascending pathways (i.e., the reticular formation and thalamo-cortical projections) [114]. Whether the signatures of glutamatergic and GABA dysfunction are caused by a direct cortical pathology or indirectly through damage arising from the brainstem structures that regulate REM sleep and then activate the cortex is still unknown. What we know is that both neuropathologic and brain imaging studies have shown alterations in several brainstem nuclei and corresponding neurotransmitters in RBD. All of these structures have widespread projections to the cerebral cortex, and therefore, perturbations of this complex network may explain the occurrence of cortical dysfunction in RBD. This has also been demonstrated by previous evidence linking cortical thinning and clinical progression in RBD [122]. Moreover, a recent study using volumetric and diffusion tensor imaging did not show significant differences in neuronal loss in the pedunculopontine nucleus according to RBD symptoms, thus suggesting that the cholinergic dysfunction of the pontine tegmentum alone is not sufficient to justify the whole symptomatology of RBD [123].

Finally, both basic and clinical evidence have indicated that RBD results from the breakdown of a broad network underlying REM sleep atonia, which provides a dynamic model of interaction between the brainstem and both rostral and caudal CNS structures [31]. Moreover, the finding of REM sleep EEG instability in RBD patients [86] and the observation that the dream content cannot be generated by the brainstem [124] stimulates further clarification on how the brainstem and the cortex interact within the complex framework of RBD pathophysiology. As a whole, this supports the view of RBD as a widespread network disorder that goes far beyond only the brainstem and the acetylcholine pathways [18,31].

### 5.2. Neurophysiology and Other Biomarkers

Aside from the neurophysiological characteristics of RBD described above, several other features are now being evaluated for consideration as markers of the disease and possibly its evolution to neurodegeneration. However, not all markers seem to have a prognostic value (although some have a role in the diagnosis of RBD) in the monitoring of its progression in severity and response to therapy or in a combination of these roles [8].

Among the neurophysiological aspects of RBD, RSWA is definitely the most easily available and least expensive, aside from its necessary value for diagnosis. RSWA is also more easily available and more cost-effective than the majority of the other markers currently under consideration which concern motor function (e.g., gait, speech, saccades, and finger tapping), cognition (e.g., Trail Making Test Part B, semantic verbal fluency, and Rey Auditory Verbal Learning Test immediate recall), olfaction (e.g., odor identification tests), ophthalmic function (e.g., the Farnsworth–Munsell 100-Hue test and optical coherence tomography), autonomic asset (e.g., questionnaires, heart rate variability, and cardiovascular reflex), biofluids (e.g., cerebrospinal fluid and serum), neuroimaging (e.g., dopamine transporter single-photon emission computed tomography, positron emission tomography with 2-deoxy-2-[fluorine-18]fluoro-D-glucose, and magnetic resonance imaging), tissue biopsy (e.g., colon, salivary glands, and skin), and genetics (e.g., *GBA* and *SNCA* 5ʹ variants) [8].

Overall, it seems reasonable to believe that, in the near future, neurophysiological biomarkers will be combined together with other biomarkers of RBD to achieve better diagnostic, prognostic, and monitoring reliability. The combined use of neurophysiological and additional more invasive or more expensive and less available biomarkers, however, might be needed, especially in the case of RBD subtyping [125], such as in the differential diagnosis between iRBD and RBD in narcolepsy, as was recently reported [5,7]. Finally, using neurophysiological features such as RSWA in combination with other types of biomarkers might have a relevant role in the identification and characterization of RBD as a prodromal form of PD [126].

## 6. Conclusions and Research Agenda

### 6.1. Final Remarks

The main points raised in this extensive narrative review of the neurophysiological aspects of RBD can be summarized as follows:

- RBD seems to modify the global neurochemical balance in areas of the central nervous system broader than the specific atonia-generating brainstem circuitry, providing a dynamic model of interaction between the brainstem and both the rostral and caudal CNS structures;
- The complex dynamical interactions between the brainstem and the other structures of the CNS are finely detected by specific neurophysiological markers, with some of them already available to both scientists and clinicians;
- Different visual scoring methods to detect and quantify RSWA have been developed and validated;
- Multiple efforts have been made to develop time-saving computerized methods for the quantification and detection of RSWA;
- EEG, TMS, and VEMP alteration studies, integrated with clinical, neuroimaging, and sleep-related data, provide novel insights into the mechanisms underlying cortical and brainstem dysfunction in RBD and might offer future therapeutic opportunities.

### 6.2. Research Agenda

Some areas of future research appear to be both needed and promising:

- Further larger studies are needed to assess which RSWA visual scoring method is most efficient and which muscle group is better suited to identify and quantify RSWA and distinguish RBD from healthy subjects;
- Although the RAI seems to be the most reliable automatic method for the scoring of RSWA and identification of RBD, additional comparative studies on larger numbers of recordings are required in order to identify and introduce the most accurate algorithm in the scoring guidelines for everyday clinical practice;
- Automated EMG quantification methods currently depend upon visual identification of REM sleep, which is harder in subjects with RSWA, as the internationally accepted criteria to score REM sleep under these circumstances should be agreed upon and required to score sleep in subjects in whom RBD is clinically suspected;
- Longitudinal studies are required to verify whether the neurophysiological abnormalities detected at the early stages of RBD by EEG, TMS, and VEMP correlate with the clinical progression of RBD;
- The real usefulness of advanced analyses of the EEG by means of innovative approaches based on artificial intelligence and artificial neural networks is still unclear and needs a deeper assessment. It is important to note that these methods also depend on the accuracy of the classification of subjects used for their training.

## Figures and Tables

**Table 1:** Accuracy of some different methods (visual and automatic) for the quantification of RSWA when used for the diagnosis of RBD.

Study	Method	Marker Type	RBD Sample Size	Accuracy
Visual methods				
McCarter et al., 2014 [53] McCarter et al., 2017 [54]	AASM [41]	Diagnostic Diagnostic	35	0.750–0.978
McCarter et al., 2014 [53] McCarter et al., 2015 [45] McCarter et al., 2017 [54]	Mayo Clinic [53]	Diagnostic Diagnostic Diagnostic	65	0.817–1.000
McCarter et al., 2019 [56]		Prognostic	60	0.563–0.925
Ferri et al., 2014 [49] Figorilli et al., 2017 [55] Figorilli et al., 2020 [57]	Montreal Group [48]	Diagnostic Diagnostic Diagnostic	270	0.597–1.000
Figorilli et al., 2017 [55] Figorilli et al., 2020 [57] Nepozitek et al., 2019 [58]	SINBAR [51]	Diagnostic Diagnostic Diagnostic	203	0.548–0.952
Automatic methods				
Ferri et al., 2013 [59] Ferri et al., 2014 [49] Figorilli et al., 2017 [55] McCarter et al., 2014 [53] McCarter et al., 2015 [45] McCarter et al., 2017 [54]	RAI [60,61]	Diagnostic Diagnostic Diagnostic Diagnostic Diagnostic Diagnostic	214	0.633–1.000
Yoshino et al., 2015 [62]	AASM [62]	Diagnostic	24	0.854
Frauscher et al., 2014 [63]	SINBAR [63]	Diagnostic	20	0.563–0.925
Cesari et al., 2019 [64]	Danish Center [64]	Diagnostic	31	0.842

AASM = American Academy of Sleep Medicine; SINBAR = Sleep Innsbruck Barcelona Group; RAI = REM Sleep Atonia Index; Danish Center = Danish Center for Sleep Medicine.

## Data Availability

Not applicable.

## References

[1] American Academy of Sleep Medicine (2014). International Classification of Sleep Disorders.

[2] Schenck C.H., Bundlie S.R., Ettinger M.G., Mahowald M.W. (1986). Chronic behavioral disorders of human REM sleep: A new category of parasomnia. Sleep.

[3] Dauvilliers Y., Schenck C.H., Postuma R.B., Iranzo A., Luppi P.H., Plazzi G., Montplaisir J., Boeve B. (2018). REM sleep behaviour disorder. Nat. Rev. Dis. Primers.

[4] Antelmi E., Pizza F., Vandi S., Neccia G., Ferri R., Bruni O., Filardi M., Cantalupo G., Liguori R., Plazzi G. (2017). The spectrum of REM sleep-related episodes in children with type 1 narcolepsy. Brain.

[5] Antelmi E., Pizza F., Donadio V., Filardi M., Sosero Y.L., Incensi A., Vandi S., Moresco M., Ferri R., Marelli S. (2019). Biomarkers for REM sleep behavior disorder in idiopathic and narcoleptic patients. Ann. Clin. Transl. Neurol..

[6] Dauvilliers Y., Jennum P., Plazzi G. (2013). Rapid eye movement sleep behavior disorder and rapid eye movement sleep without atonia in narcolepsy. Sleep Med..

[7] Antelmi E., Pizza F., Franceschini C., Ferri R., Plazzi G. (2020). REM sleep behavior disorder in narcolepsy: A secondary form or an intrinsic feature?. Sleep Med. Rev..

[8] Miglis M.G., Adler C.H., Antelmi E., Arnaldi D., Baldelli L., Boeve B.F., Cesari M., Dall’Antonia I., Diederich N.J., Doppler K. (2021). Biomarkers of conversion to α-synucleinopathy in isolated rapid-eye-movement sleep behaviour disorder. Lancet Neurol..

[9] Cesari M., Heidbreder A., St Louis E.K., Sixel-Doring F., Bliwise D.L., Baldelli L., Bes F., Fantini M.L., Iranzo A., Knudsen-Heier S. (2021). Video-polysomnography procedures for diagnosis of rapid eye movement sleep behavior disorder (RBD) and the identification of its prodromal stages: Guidelines from the International RBD Study Group. Sleep.

[10] Manni R., Ratti P.L., Terzaghi M. (2011). Secondary “incidental” REM sleep behavior disorder: Do we ever think of it?. Sleep Med..

[11] Zambelis T., Paparrigopoulos T., Soldatos C.R. (2002). REM sleep behaviour disorder associated with a neurinoma of the left pontocerebellar angle. J. Neurol. Neurosurg. Psychiatry.

[12] Provini F., Vetrugno R., Pastorelli F., Lombardi C., Plazzi G., Marliani A.F., Lugaresi E., Montagna P. (2004). Status dissociatus after surgery for tegmental ponto-mesencephalic cavernoma: A state-dependent disorder of motor control during sleep. Mov. Disord. Off. J. Mov. Disord. Soc..

[13] McKenna D., Peever J. (2017). Degeneration of rapid eye movement sleep circuitry underlies rapid eye movement sleep behavior disorder. Mov. Disord. Off. J. Mov. Disord. Soc..

[14] Jouvet M. (1962). Recherches sur les structures nerveuses et les mecanismes responsables des differentes phases du sommeil physiologique. Arch. Ital. Biol..

[15] Sakai K., Koyama Y. (1996). Are there cholinergic and non-cholinergic paradoxical sleep-on neurones in the pons?. Neuroreport.

[16] Vanni-Mercier G., Sakai K., Lin J.S., Jouvet M. (1989). Mapping of cholinoceptive brainstem structures responsible for the generation of paradoxical sleep in the cat. Arch. Ital. Biol..

[17] Clement O., Sapin E., Berod A., Fort P., Luppi P.H. (2011). Evidence that neurons of the sublaterodorsal tegmental nucleus triggering paradoxical (REM) sleep are glutamatergic. Sleep.

[18] Valencia Garcia S., Libourel P.A., Lazarus M., Grassi D., Luppi P.H., Fort P. (2017). Genetic inactivation of glutamate neurons in the rat sublaterodorsal tegmental nucleus recapitulates REM sleep behaviour disorder. Brain.

[19] Luppi P.H., Fort P. (2019). Sleep-wake physiology. Handb. Clin. Neurol..

[20] Varin C., Luppi P.H., Fort P. (2018). Melanin-concentrating hormone-expressing neurons adjust slow-wave sleep dynamics to catalyze paradoxical (REM) sleep. Sleep.

[21] Sapin E., Lapray D., Berod A., Goutagny R., Leger L., Ravassard P., Clement O., Hanriot L., Fort P., Luppi P.H. (2009). Localization of the brainstem GABAergic neurons controlling paradoxical (REM) sleep. PLoS ONE.

[22] Hayashi Y., Kashiwagi M., Yasuda K., Ando R., Kanuka M., Sakai K., Itohara S. (2015). Cells of a common developmental origin regulate REM/non-REM sleep and wakefulness in mice. Science.

[23] Luppi P.H., Clement O., Sapin E., Gervasoni D., Peyron C., Leger L., Salvert D., Fort P. (2011). The neuronal network responsible for paradoxical sleep and its dysfunctions causing narcolepsy and rapid eye movement (REM) behavior disorder. Sleep Med. Rev..

[24] McGinty D.J., Harper R.M. (1976). Dorsal raphe neurons: Depression of firing during sleep in cats. Brain Res..

[25] Nakamura Y., Goldberg L.J., Chandler S.H., Chase M.H. (1978). Intracellular analysis of trigeminal motoneuron activity during sleep in the cat. Science.

[26] Brooks P.L., Peever J.H. (2012). Identification of the transmitter and receptor mechanisms responsible for REM sleep paralysis. J. Neurosci..

[27] Soja P.J., Lopez-Rodriguez F., Morales F.R., Chase M.H. (1991). The postsynaptic inhibitory control of lumbar motoneurons during the atonia of active sleep: Effect of strychnine on motoneuron properties. J. Neurosci..

[28] Burgess C., Lai D., Siegel J., Peever J. (2008). An endogenous glutamatergic drive onto somatic motoneurons contributes to the stereotypical pattern of muscle tone across the sleep-wake cycle. J. Neurosci..

[29] Lai Y.Y., Kodama T., Siegel J.M. (2001). Changes in monoamine release in the ventral horn and hypoglossal nucleus linked to pontine inhibition of muscle tone: An in vivo microdialysis study. J. Neurosci..

[30] Fenik V.B., Davies R.O., Kubin L. (2005). REM sleep-like atonia of hypoglossal (XII) motoneurons is caused by loss of noradrenergic and serotonergic inputs. Am. J. Respir. Crit. Care Med..

[31] Peever J., Luppi P.H., Montplaisir J. (2014). Breakdown in REM sleep circuitry underlies REM sleep behavior disorder. Trends Neurosci..

[32] Fraigne J.J., Torontali Z.A., Snow M.B., Peever J.H. (2015). REM Sleep at its Core—Circuits, Neurotransmitters, and Pathophysiology. Front. Neurol..

[33] Boissard R., Gervasoni D., Schmidt M.H., Barbagli B., Fort P., Luppi P.H. (2002). The rat ponto-medullary network responsible for paradoxical sleep onset and maintenance: A combined microinjection and functional neuroanatomical study. Eur. J. Neurosci..

[34] Peever J., Fuller P.M. (2016). Neuroscience: A Distributed Neural Network Controls REM Sleep. Curr. Biol..

[35] Torontali Z.A., Grace K.P., Horner R.L., Peever J.H. (2014). Cholinergic involvement in control of REM sleep paralysis. J. Physiol..

[36] Weng F.J., Williams R.H., Hawryluk J.M., Lu J., Scammell T.E., Saper C.B., Arrigoni E. (2014). Carbachol excites sublaterodorsal nucleus neurons projecting to the spinal cord. J. Physiol..

[37] Krenzer M., Anaclet C., Vetrivelan R., Wang N., Vong L., Lowell B.B., Fuller P.M., Lu J. (2011). Brainstem and spinal cord circuitry regulating REM sleep and muscle atonia. PLoS ONE.

[38] Iranzo A., Tolosa E., Gelpi E., Molinuevo J.L., Valldeoriola F., Serradell M., Sanchez-Valle R., Vilaseca I., Lomena F., Vilas D. (2013). Neurodegenerative disease status and post-mortem pathology in idiopathic rapid-eye-movement sleep behaviour disorder: An observational cohort study. Lancet Neurol..

[39] Ehrminger M., Latimier A., Pyatigorskaya N., Garcia-Lorenzo D., Leu-Semenescu S., Vidailhet M., Lehericy S., Arnulf I. (2016). The coeruleus/subcoeruleus complex in idiopathic rapid eye movement sleep behaviour disorder. Brain.

[40] Garcia-Lorenzo D., Longo-Dos Santos C., Ewenczyk C., Leu-Semenescu S., Gallea C., Quattrocchi G., Pita Lobo P., Poupon C., Benali H., Arnulf I. (2013). The coeruleus/subcoeruleus complex in rapid eye movement sleep behaviour disorders in Parkinson’s disease. Brain.

[41] Berry R.B., Quan S.F., Abreu A.R., Bibbs M.L., DelRosso L., Harding S.M., Mao M.-M., Plante D.T., Pressman M.R., Troester M.R. (2020). The AASM Manual for the Scoring of Sleep and Associated Events: Rules, Terminology and Technical Specifications, Version 2.6.

[42] Hoque R., Chesson A.L. (2010). Pharmacologically induced/exacerbated restless legs syndrome, periodic limb movements of sleep, and REM behavior disorder/REM sleep without atonia: Literature review, qualitative scoring, and comparative analysis. J. Clin. Sleep Med. JCSM Off. Publ. Am. Acad. Sleep Med..

[43] Postuma R.B., Gagnon J.F., Tuineaig M., Bertrand J.A., Latreille V., Desjardins C., Montplaisir J.Y. (2013). Antidepressants and REM sleep behavior disorder: Isolated side effect or neurodegenerative signal?. Sleep.

[44] Lee K., Baron K., Soca R., Attarian H. (2016). The Prevalence and Characteristics of REM Sleep without Atonia (RSWA) in Patients Taking Antidepressants. J. Clin. Sleep Med..

[45] McCarter S.J., St Louis E.K., Sandness D.J., Arndt K., Erickson M., Tabatabai G., Boeve B.F., Silber M.H. (2015). Antidepressants Increase REM Sleep Muscle Tone in Patients with and without REM Sleep Behavior Disorder. Sleep.

[46] Iranzo A., Santamaria J. (1999). Bisoprolol-induced rapid eye movement sleep behavior disorder. Am. J. Med..

[47] Ferri R., Mogavero M.P., Bruni O., Plazzi G., Schenck C.H., DelRosso L.M. (2021). Increased Chin Muscle Tone during All Sleep Stages in Children Taking SSRI Antidepressants and in Children with Narcolepsy Type 1. Sleep.

[48] Lapierre O., Montplaisir J. (1992). Polysomnographic features of REM sleep behavior disorder: Development of a scoring method. Neurology.

[49] Ferri R., Gagnon J.F., Postuma R.B., Rundo F., Montplaisir J.Y. (2014). Comparison between an automatic and a visual scoring method of the chin muscle tone during rapid eye movement sleep. Sleep Med..

[50] Montplaisir J., Gagnon J.F., Fantini M.L., Postuma R.B., Dauvilliers Y., Desautels A., Rompre S., Paquet J. (2010). Polysomnographic diagnosis of idiopathic REM sleep behavior disorder. Mov. Disord. Off. J. Mov. Disord. Soc..

[51] Frauscher B., Iranzo A., Gaig C., Gschliesser V., Guaita M., Raffelseder V., Ehrmann L., Sola N., Salamero M., Tolosa E. (2012). Normative EMG values during REM sleep for the diagnosis of REM sleep behavior disorder. Sleep.

[52] Bliwise D.L., Rye D.B. (2008). Elevated PEM (phasic electromyographic metric) rates identify rapid eye movement behavior disorder patients on nights without behavioral abnormalities. Sleep.

[53] McCarter S.J., St Louis E.K., Duwell E.J., Timm P.C., Sandness D.J., Boeve B.F., Silber M.H. (2014). Diagnostic thresholds for quantitative REM sleep phasic burst duration, phasic and tonic muscle activity, and REM atonia index in REM sleep behavior disorder with and without comorbid obstructive sleep apnea. Sleep.

[54] McCarter S.J., St Louis E.K., Sandness D.J., Duwell E.J., Timm P.C., Boeve B.F., Silber M.H. (2017). Diagnostic REM sleep muscle activity thresholds in patients with idiopathic REM sleep behavior disorder with and without obstructive sleep apnea. Sleep Med..

[55] Figorilli M., Ferri R., Zibetti M., Beudin P., Puligheddu M., Lopiano L., Cicolin A., Durif F., Marques A., Fantini M.L. (2017). Comparison Between Automatic and Visual Scorings of REM Sleep Without Atonia for the Diagnosis of REM Sleep Behavior Disorder in Parkinson Disease. Sleep.

[56] McCarter S.J., Sandness D.J., McCarter A.R., Feemster J.C., Teigen L.N., Timm P.C., Boeve B.F., Silber M.H., St Louis E.K. (2019). REM sleep muscle activity in idiopathic REM sleep behavior disorder predicts phenoconversion. Neurology.

[57] Figorilli M., Marques A.R., Meloni M., Zibetti M., Pereira B., Lambert C., Puligheddu M., Cicolin A., Lopiano L., Durif F. (2020). Diagnosing REM sleep behavior disorder in Parkinson’s disease without a gold standard: A latent-class model study. Sleep.

[58] Nepozitek J., Dostalova S., Dusek P., Kemlink D., Prihodova I., Ibarburu Lorenzo Y.L.V., Friedrich L., Bezdicek O., Nikolai T., Perinova P. (2019). Simultaneous tonic and phasic REM sleep without atonia best predicts early phenoconversion to neurodegenerative disease in idiopathic REM sleep behavior disorder. Sleep.

[59] Ferri R., Marelli S., Cosentino F.I., Rundo F., Ferini-Strambi L., Zucconi M. (2013). Night-to-night variability of automatic quantitative parameters of the chin EMG amplitude (Atonia Index) in REM sleep behavior disorder. J. Clin. Sleep Med. JCSM Off. Publ. Am. Acad. Sleep Med..

[60] Ferri R., Manconi M., Plazzi G., Bruni O., Vandi S., Montagna P., Ferini-Strambi L., Zucconi M. (2008). A quantitative statistical analysis of the submentalis muscle EMG amplitude during sleep in normal controls and patients with REM sleep behavior disorder. J. Sleep Res..

[61] Ferri R., Rundo F., Manconi M., Plazzi G., Bruni O., Oldani A., Ferini-Strambi L., Zucconi M. (2010). Improved computation of the atonia index in normal controls and patients with REM sleep behavior disorder. Sleep Med..

[62] Yoshino K., Kimura N., Iyama A., Sakoda S. (2015). Automatic Quantification of Muscular Activity in Rapid Eye Movement Sleep. Adv. Biomed. Eng..

[63] Frauscher B., Gabelia D., Biermayr M., Stefani A., Hackner H., Mitterling T., Poewe W., Hogl B. (2014). Validation of an integrated software for the detection of rapid eye movement sleep behavior disorder. Sleep.

[64] Cesari M., Christensen J.A.E., Sorensen H.B.D., Jennum P., Mollenhauer B., Muntean M.L., Trenkwalder C., Sixel-Doring F. (2019). External validation of a data-driven algorithm for muscular activity identification during sleep. J. Sleep Res..

[65] Frauscher B., Ehrmann L., Hogl B. (2013). Defining muscle activities for assessment of rapid eye movement sleep behavior disorder: From a qualitative to a quantitative diagnostic level. Sleep Med..

[66] Neikrug A.B., Ancoli-Israel S. (2012). Diagnostic tools for REM sleep behavior disorder. Sleep Med. Rev..

[67] Ferri R., Bruni O., Fulda S., Zucconi M., Plazzi G. (2012). A quantitative analysis of the submentalis muscle electromyographic amplitude during rapid eye movement sleep across the lifespan. J. Sleep Res..

[68] Ferri R., Fulda S., Cosentino F.I., Pizza F., Plazzi G. (2012). A preliminary quantitative analysis of REM sleep chin EMG in Parkinson’s disease with or without REM sleep behavior disorder. Sleep Med..

[69] Ferri R., Franceschini C., Zucconi M., Vandi S., Poli F., Bruni O., Cipolli C., Montagna P., Plazzi G. (2008). Searching for a marker of REM sleep behavior disorder: Submentalis muscle EMG amplitude analysis during sleep in patients with narcolepsy/cataplexy. Sleep.

[70] Olesen A.N., Cesari M., Christensen J.A.E., Sorensen H.B.D., Mignot E., Jennum P. (2018). A comparative study of methods for automatic detection of rapid eye movement abnormal muscular activity in narcolepsy. Sleep Med..

[71] Burns J.W., Consens F.B., Little R.J., Angell K.J., Gilman S., Chervin R.D. (2007). EMG variance during polysomnography as an assessment for REM sleep behavior disorder. Sleep.

[72] Mayer G., Kesper K., Ploch T., Canisius S., Penzel T., Oertel W., Stiasny-Kolster K. (2008). Quantification of tonic and phasic muscle activity in REM sleep behavior disorder. J. Clin. Neurophysiol. Off. Publ. Am. Electroencephalogr. Soc..

[73] Kempfner J., Sorensen H.B., Nikolic M., Jennum P. (2014). Early automatic detection of Parkinson’s disease based on sleep recordings. J. Clin. Neurophysiol. Off. Publ. Am. Electroencephalogr. Soc..

[74] Frandsen R., Nikolic M., Zoetmulder M., Kempfner L., Jennum P. (2015). Analysis of automated quantification of motor activity in REM sleep behaviour disorder. J. Sleep Res..

[75] Jeppesen J., Otto M., Frederiksen Y., Hansen A.K., Fedorova T.D., Knudsen K., Nahimi A., Brooks D.J., Borghammer P., Sommerauer M. (2018). Observations on muscle activity in REM sleep behavior disorder assessed with a semi-automated scoring algorithm. Clin. Neurophysiol. Off. J. Int. Fed. Clin. Neurophysiol..

[76] Fairley J.A., Georgoulas G., Stylios C.D., Vachtsevanos G., Rye D.B., Bliwise D.L. Phasic Electromyographic Metric detection based on wavelet analysis. Proceedings of the 2011 19th Mediterranean Conference on Control & Automation (MED).

[77] Milerska I., Kremen V., Gerla V., St Louis E.K., Lhotska L. (2019). Semi-automated Detection of Polysomnographic REM Sleep without Atonia (RSWA) in REM Sleep Behavioral Disorder. Biomed. Signal Process. Control.

[78] Cooray N., Andreotti F., Lo C., Symmonds M., Hu M.T.M., De Vos M. (2019). Detection of REM sleep behaviour disorder by automated polysomnography analysis. Clin. Neurophysiol. Off. J. Int. Fed. Clin. Neurophysiol..

[79] Cooray N., Andreotti F., Lo C., Symmonds M., Hu M.T.M., De Vos M. (2021). Proof of concept: Screening for REM sleep behaviour disorder with a minimal set of sensors. Clin. Neurophysiol. Off. J. Int. Fed. Clin. Neurophysiol..

[80] Cesari M., Christensen J.A.E., Sixel-Doring F., Trenkwalder C., Mayer G., Oertel W.H., Jennum P., Sorensen H.B.D. (2019). Validation of a new data-driven automated algorithm for muscular activity detection in REM sleep behavior disorder. J. Neurosci. Methods.

[81] Cesari M., Christensen J.A.E., Muntean M.L., Mollenhauer B., Sixel-Doring F., Sorensen H.B.D., Trenkwalder C., Jennum P. (2021). A data-driven system to identify REM sleep behavior disorder and to predict its progression from the prodromal stage in Parkinson’s disease. Sleep Med..

[82] Cesari M., Christensen J.A.E., Kempfner L., Olesen A.N., Mayer G., Kesper K., Oertel W.H., Sixel-Doring F., Trenkwalder C., Sorensen H.B.D. (2018). Comparison of computerized methods for rapid eye movement sleep without atonia detection. Sleep.

[83] Aarsland D. (2016). Cognitive impairment in Parkinson’s disease and dementia with Lewy bodies. Parkinsonism Relat. Disord..

[84] Ferini-Strambi L., Fasiello E., Sforza M., Salsone M., Galbiati A. (2019). Neuropsychological, electrophysiological, and neuroimaging biomarkers for REM behavior disorder. Expert Rev. Neurother..

[85] Valomon A., Riedner B.A., Jones S.G., Nakamura K.P., Tononi G., Plante D.T., Benca R.M., Boly M. (2021). A high-density electroencephalography study reveals abnormal sleep homeostasis in patients with rapid eye movement sleep behavior disorder. Sci. Rep..

[86] Ferri R., Rundo F., Silvani A., Zucconi M., Bruni O., Ferini-Strambi L., Plazzi G., Manconi M. (2017). REM Sleep EEG Instability in REM Sleep Behavior Disorder and Clonazepam Effects. Sleep.

[87] Parrino L., Ferri R., Bruni O., Terzano M.G. (2012). Cyclic alternating pattern (CAP): The marker of sleep instability. Sleep Med. Rev..

[88] Ferri R., Zucconi M., Marelli S., Plazzi G., Schenck C.H., Ferini-Strambi L. (2013). Effects of long-term use of clonazepam on nonrapid eye movement sleep patterns in rapid eye movement sleep behavior disorder. Sleep Med..

[89] Kutlu A., Iseri P., Selekler M., Benbir G., Karadeniz D. (2013). Cyclic alternating pattern analysis in REM sleep behavior disorder. Sleep Breath. Schlaf Atm..

[90] Melpignano A., Parrino L., Santamaria J., Gaig C., Trippi I., Serradell M., Mutti C., Ricco M., Iranzo A. (2019). Isolated rapid eye movement sleep behavior disorder and cyclic alternating pattern: Is sleep microstructure a predictive parameter of neurodegeneration?. Sleep.

[91] O’Reilly C., Godin I., Montplaisir J., Nielsen T. (2015). REM sleep behaviour disorder is associated with lower fast and higher slow sleep spindle densities. J. Sleep Res..

[92] Christensen J.A., Kempfner J., Zoetmulder M., Leonthin H.L., Arvastson L., Christensen S.R., Sorensen H.B., Jennum P. (2014). Decreased sleep spindle density in patients with idiopathic REM sleep behavior disorder and patients with Parkinson’s disease. Clin. Neurophysiol. Off. J. Int. Fed. Clin. Neurophysiol..

[93] Ruffini G., Ibañez D., Castellano M., Dubreuil-Vall L., Soria-Frisch A., Postuma R., Gagnon J.F., Montplaisir J. (2019). Deep Learning With EEG Spectrograms in Rapid Eye Movement Behavior Disorder. Front. Neurol..

[94] Agarwal S., Koch G., Hillis A.E., Huynh W., Ward N.S., Vucic S., Kiernan M.C. (2019). Interrogating cortical function with transcranial magnetic stimulation: Insights from neurodegenerative disease and stroke. J. Neurol. Neurosurg. Psychiatry.

[95] Di Lazzaro V., Bella R., Benussi A., Bologna M., Borroni B., Capone F., Chen K.S., Chen R., Chistyakov A.V., Classen J. (2021). Diagnostic contribution and therapeutic perspectives of transcranial magnetic stimulation in dementia. Clin. Neurophysiol. Off. J. Int. Fed. Clin. Neurophysiol..

[96] Nardone R., Sebastianelli L., Versace V., Brigo F., Golaszewski S., Pucks-Faes E., Saltuari L., Trinka E. (2020). Effects of repetitive transcranial magnetic stimulation in subjects with sleep disorders. Sleep Med..

[97] Lanza G. (2020). Repetitive TMS for sleep disorders: Are we ready?. Sleep Med..

[98] Lanza G. (2021). Repetitive TMS for the “cognitive tsunami” of sleep deprivation. Sleep Med..

[99] Cantone M., Lanza G., Vinciguerra L., Puglisi V., Ricceri R., Fisicaro F., Vagli C., Bella R., Ferri R., Pennisi G. (2019). Age, Height, and Sex on Motor Evoked Potentials: Translational Data From a Large Italian Cohort in a Clinical Environment. Front. Hum. Neurosci..

[100] Fisicaro F., Lanza G., Cantone M., Ferri R., Pennisi G., Nicoletti A., Zappia M., Bella R., Pennisi M. (2020). Clinical and Electrophysiological Hints to TMS in De Novo Patients with Parkinson’s Disease and Progressive Supranuclear Palsy. J. Pers. Med..

[101] Nardone R., Brigo F., Versace V., Holler Y., Tezzon F., Saltuari L., Trinka E., Sebastianelli L. (2017). Cortical afferent inhibition abnormalities reveal cholinergic dysfunction in Parkinson’s disease: A reappraisal. J. Neural Transm..

[102] Lanza G., Ferri R. (2019). The neurophysiology of hyperarousal in restless legs syndrome: Hints for a role of glutamate/GABA. Adv. Pharmacol..

[103] Lanza G., Lanuzza B., Arico D., Cantone M., Cosentino F.I.I., Bella R., Pennisi G., Ferri R., Pennisi M. (2018). Impaired short-term plasticity in restless legs syndrome: A pilot rTMS study. Sleep Med..

[104] Magalhaes S.C., Kaelin-Lang A., Sterr A., do Prado G.F., Eckeli A.L., Conforto A.B. (2015). Transcranial magnetic stimulation for evaluation of motor cortical excitability in restless legs syndrome/Willis-Ekbom disease. Sleep Med..

[105] Cantone M., Lanza G., Ranieri F., Opie G.M., Terranova C. (2021). Editorial: Non-invasive Brain Stimulation in the Study and Modulation of Metaplasticity in Neurological Disorders. Front. Neurol..

[106] Lanza G., Scalise A. (2020). The present and the future of Transcranial Magnetic Stimulation in Restless Legs Syndrome. Sleep Med..

[107] Bares M., Kanovsky P., Klajblova H., Rektor I. (2003). Intracortical inhibition and facilitation are impaired in patients with early Parkinson’s disease: A paired TMS study. Eur. J. Neurol..

[108] Lefaucheur J.P. (2005). Motor cortex dysfunction revealed by cortical excitability studies in Parkinson’s disease: Influence of antiparkinsonian treatment and cortical stimulation. Clin. Neurophysiol. Off. J. Int. Fed. Clin. Neurophysiol..

[109] Vacherot F., Attarian S., Vaugoyeau M., Azulay J.P. (2010). A motor cortex excitability and gait analysis on Parkinsonian patients. Mov. Disord. Off. J. Mov. Disord. Soc..

[110] Kacar A., Filipovic S.R., Kresojevic N., Milanovic S.D., Ljubisavljevic M., Kostic V.S., Rothwell J.C. (2013). History of exposure to dopaminergic medication does not affect motor cortex plasticity and excitability in Parkinson’s disease. Clin. Neurophysiol. Off. J. Int. Fed. Clin. Neurophysiol..

[111] Leon-Sarmiento F.E., Rizzo-Sierra C.V., Bayona E.A., Bayona-Prieto J., Doty R.L., Bara-Jimenez W. (2013). Novel mechanisms underlying inhibitory and facilitatory transcranial magnetic stimulation abnormalities in Parkinson’s disease. Arch. Med Res..

[112] Kobayashi M., Ohira T., Mihara B., Fujimaki T. (2016). Changes in intracortical inhibition and clinical symptoms after STN-DBS in Parkinson’s disease. Clin. Neurophysiol. Off. J. Int. Fed. Clin. Neurophysiol..

[113] Fernandez-Lago H., Bello O., Mora-Cerda F., Montero-Camara J., Fernandez-Del-Olmo M.A. (2017). Treadmill Walking Combined With Anodal Transcranial Direct Current Stimulation in Parkinson Disease: A Pilot Study of Kinematic and Neurophysiological Effects. Am. J. Phys. Med. Rehabil..

[114] Nardone R., Bergmann J., Kunz A., Christova M., Brigo F., Tezzon F., Trinka E., Golaszewski S. (2012). Cortical afferent inhibition is reduced in patients with idiopathic REM sleep behavior disorder and cognitive impairment: A TMS study. Sleep Med..

[115] Nardone R., Bergmann J., Brigo F., Christova M., Kunz A., Seidl M., Tezzon F., Trinka E., Golaszewski S. (2013). Functional evaluation of central cholinergic circuits in patients with Parkinson’s disease and REM sleep behavior disorder: A TMS study. J. Neural Transm..

[116] Lanza G., Arico D., Lanuzza B., Cosentino F.I.I., Tripodi M., Giardina F., Bella R., Puligheddu M., Pennisi G., Ferri R. (2020). Facilitatory/inhibitory intracortical imbalance in REM sleep behavior disorder: Early electrophysiological marker of neurodegeneration?. Sleep.

[117] Kohyama J. (2000). REM sleep atonia: Responsible brain regions, quantification, and clinical implication. Brain Dev..

[118] Magnano I., Pes G.M., Cabboi M.P., Pilurzi G., Ginatempo F., Achene A., Salis A., Conti M., Deriu F. (2016). Comparison of brainstem reflex recordings and evoked potentials with clinical and MRI data to assess brainstem dysfunction in multiple sclerosis: A short-term follow-up. Neurol. Sci. Off. J. Ital. Neurol. Soc. Ital. Soc. Clin. Neurophysiol..

[119] De Natale E.R., Ginatempo F., Laccu I., Figorilli M., Manca A., Mercante B., Puligheddu M., Deriu F. (2018). Vestibular Evoked Myogenic Potentials Are Abnormal in Idiopathic REM Sleep Behavior Disorder. Front. Neurol..

[120] De Natale E.R., Ginatempo F., Paulus K.S., Pes G.M., Manca A., Tolu E., Agnetti V., Deriu F. (2015). Abnormalities of vestibular-evoked myogenic potentials in idiopathic Parkinson’s disease are associated with clinical evidence of brainstem involvement. Neurol. Sci. Off. J. Ital. Neurol. Soc. Ital. Soc. Clin. Neurophysiol..

[121] Puligheddu M., Figorilli M., Serra A., Laccu I., Congiu P., Tamburrino L., de Natale E.R., Ginatempo F., Deriu F., Loi G. (2019). REM Sleep without atonia correlates with abnormal vestibular-evoked myogenic potentials in isolated REM sleep behavior disorder. Sleep.

[122] Pereira J.B., Weintraub D., Chahine L., Aarsland D., Hansson O., Westman E. (2019). Cortical thinning in patients with REM sleep behavior disorder is associated with clinical progression. NPJ Parkinson’s Dis..

[123] Dugger B.N., Murray M.E., Boeve B.F., Parisi J.E., Benarroch E.E., Ferman T.J., Dickson D.W. (2012). Neuropathological analysis of brainstem cholinergic and catecholaminergic nuclei in relation to rapid eye movement (REM) sleep behaviour disorder. Neuropathol. Appl. Neurobiol..

[124] Cipolli C., Ferrara M., De Gennaro L., Plazzi G. (2017). Beyond the neuropsychology of dreaming: Insights into the neural basis of dreaming with new techniques of sleep recording and analysis. Sleep Med. Rev..

[125] Iranzo A., Santamaria J., Rye D.B., Valldeoriola F., Marti M.J., Munoz E., Vilaseca I., Tolosa E. (2005). Characteristics of idiopathic REM sleep behavior disorder and that associated with MSA and PD. Neurology.

[126] Berg D., Borghammer P., Fereshtehnejad S.M., Heinzel S., Horsager J., Schaeffer E., Postuma R.B. (2021). Prodromal Parkinson disease subtypes—Key to understanding heterogeneity. Nat. Rev. Neurol..

